# Contrast-enhanced ultrasound (CEUS) in urgent and emergent pediatric conditions: a comprehensive review

**DOI:** 10.1007/s10140-025-02432-4

**Published:** 2026-01-21

**Authors:** Ju Hee Ahn, Preet K. Sandhu, Natasha Honda, Amy Crumb, Reza J. Daugherty

**Affiliations:** 1 Department of Radiology and Medical Imaging, University of Virginia Health, 1215 Lee Street, Charlottesville, 22908 VA USA; 2 Department of Radiology, Le Bonheur Children’s Hospital, University of Tennessee Health Science Center, 848 Adams Avenue, Memphis, 38103 TN USA; 3 Department of Radiology, Children’s Hospital Los Angeles, 4650 Sunset Boulevard, Los Angeles, 90027 CA USA

**Keywords:** Contrast-enhanced ultrasound, Ultrasound, Blunt abdominal trauma, Pediatric, Emergency medicine

## Abstract

Approximately 30 million children visit emergency departments in the United States annually presenting with a wide spectrum of urgent and emergent traumatic and non-traumatic conditions. Prompt and accurate imaging evaluation is critical for diagnosis, risk stratification, and appropriate management. Contrast-enhanced ultrasound (CEUS) lacks ionizing radiation and has therefore emerged as a reliable and accurate imaging modality for assessing many pediatric conditions. Building on conventional ultrasound (US), CEUS uses ultrasound contrast agents (UCA) to enhance visualization of parenchymal perfusion and microvascularity and demonstrates diagnostic sensitivity comparable to that of contrast-enhanced computed tomography (CT) for several clinical scenarios. This paper reviews the expanding utilization of CEUS in urgent and emergent pediatric care, with particular emphasis on blunt abdominal trauma, and describes the characteristic imaging features of both traumatic and non-traumatic pathologies. Additionally, the basic principles, techniques, and limitations of CEUS are briefly addressed.

**Graphical abstract:**

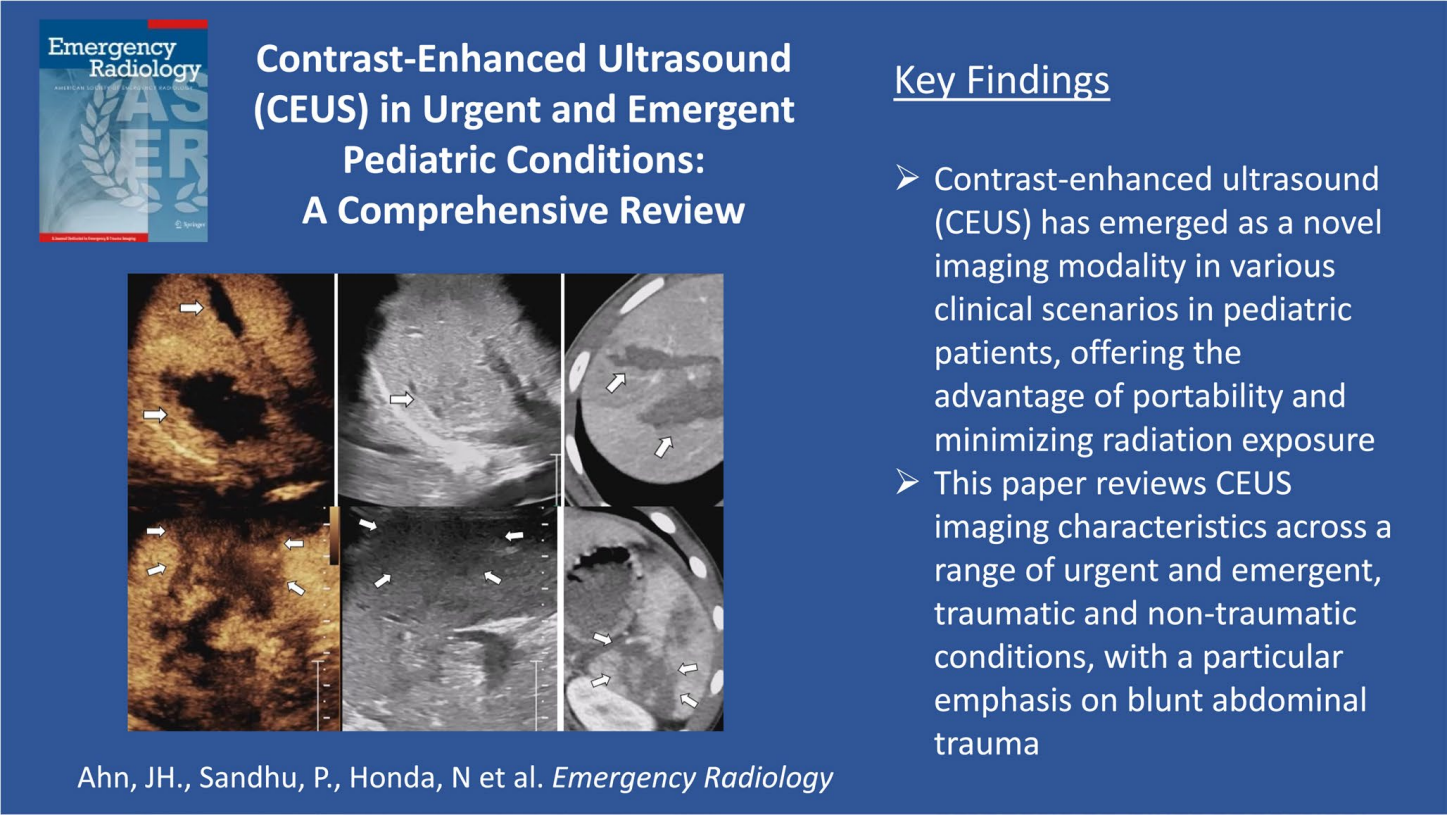

## Introduction

Children account for approximately 20% of all emergency department (ED) visits in the United States. Trauma is the leading cause of pediatric mortality in pediatric emergency medicine. Additionally, a variety of non-traumatic urgent and emergent illnesses are frequently encountered in the ED and hospital setting [1]. Ultrasound (US) is a widely accessible and safe imaging modality that does not involve ionizing radiation and can be performed relatively easily at the bedside. Because of these advantages, US is the first-line imaging modality for evaluation of many urgent and emergent conditions. However, conventional gray-scale US and Doppler US have inherent limitations, particularly for evaluating parenchymal perfusion and microvascularity.

Contrast-enhanced ultrasound (CEUS) has become an integral component of pediatric clinical practice worldwide, facilitated by advancements in ultrasound contrast agents (UCA). Due to their purely intravascular properties, UCAs enable real-time evaluation of solid organ parenchymal enhancement across all vascular phases, thereby complementing the diagnostic capabilities of conventional US. *Menichini et al.* demonstrated that CEUS exhibits greater sensitivity and accuracy than baseline US and comparable sensitivity to computed tomography (CT) in identifying and characterizing traumatic solid organ injuries in pediatric patients [2].

This paper presents a comprehensive review of the diverse traumatic and non-traumatic applications of CEUS, with a particular emphasis on its role in its most widely studied application – assessing blunt abdominal trauma. We also discuss the basic principles, safety, and limitations of CEUS.

## Contrast-enhanced ultrasound

### UCA and basic principles

UCAs consist of gas-filled microbubbles encapsulated by shells composed of various substances, including galactose, lipids, and albumin. Different UCAs vary in shell composition, size, and gas core components [3]. The encapsulating shell provides stability and contributes to the resonant properties of the microbubbles. Once administered intravenously, microbubbles are distributed throughout vessels and organs, enabling real-time evaluation of tissue perfusion. When exposed to high-energy ultrasound impulses, characterized by a high mechanical index (MI), the microbubbles rupture [4–6].

The latest generation of microbubbles exhibits enhanced stability, allowing for improved contrast and prolonged observation of blood flow within the area of interest. Lumason (Bracco Diagnostic Inc., Monroe Township, NJ) has been approved by the United States Food and Drug Administration (FDA) for indications of echocardiography, focal liver lesion characterization, and intravesical use for evaluating vesicoureteral reflux. It is marketed as SonoVue (Bracco Imaging Spa, Milan, Italy) outside of the United States. Definity (Lantheus, North Billerica, MA) and Optison (GE Healthcare, Princeton, NJ) were approved for echocardiography by the FDA. Other UCAs and CEUS applications in pediatric imaging remain off-label in the United States but are widely used for indications such as renal lesion characterization or bowel imaging in inflammatory bowel disease.

## UCA dose and safety

The FDA-approved intravenous (IV) dose of Lumason/SonoVue is 0.03 mL/kg, with a maximum single bolus injection limit of 2.4 mL, which can be repeated one time during the same examination. When a second bolus injection is needed, an interval of approximately 10–15 min should be allowed to ensure that microbubbles from the prior injection have disappeared or have been substantially diminished [7]. The FDA-approved bolus dose of Definity is 3µL/kg, and the maximum dose is two bolus injection administered 30 min apart. The FDA-recommended dose of Optison for pediatric patients is based on weight adjust dosing. For scrotal imaging, pediatric dosing guidelines are not established: however, a double-dose approach (one dose per testis) has been considered appropriate by some authors. The use of high-frequency transducers necessitates an increased dose of UCA in certain situations [8, 9].

Children exhibit lower rates of contrast reactions to UCAs compared to adults, with a notably low incidence of complications [6, 10]. The most common adverse reactions associated with UCAs include headache and nausea, which occur at a rate of less than 0.5%. A review of 57 studies involving 4,518 children with 4,906 CEUS examinations reported a 1.20% incidence of non-serious adverse events and a 0.22% incidence of serious adverse events, with hypersensitivity reactions accounting for the majority of severe cases [11].

The contraindications for the use of UCAs in pediatric patients are aligned with those established for adults. These include a history of hypersensitivity to UCAs or their components, severe pulmonary hypertension, and uncontrolled systemic hypertension. Definity should be used with caution in patients with sickle cell disease and discontinued if new or worsening pain occurs. All precautions for the prompt recognition and management of potential adverse reactions should be performed prior to administering a UCA, and examinations should be optimally performed within a dedicated hospital-based department where appropriate expertise is readily available [12].

## General technique

A gray-scale and color Doppler US of the area of interest should be performed before CEUS to assess anatomical details. The UCA is then administered using a three-way stopcock, followed by a normal saline flush for optimal delivery. To ensure the transducer remains within the desired area of interest, a dual-display option is utilized, allowing simultaneous visualization of the B-mode as a reference alongside the contrast image. CEUS examination should be conducted using a low MI (0.05–0.2) to prevent microbubble burst and ensure optimal image quality [3, 7, 13]. In addition, the US scan parameters should remain unchanged throughout the examination and any follow-up examinations, particularly in cases where quantitative evaluation is desired.

## Clinical applications of CEUS

### Blunt abdominal trauma

Trauma is a leading cause of morbidity and mortality in children and adolescents, with blunt abdominal trauma (BAT) accounting for 80–90% of pediatric trauma cases [14]. Due to their smaller abdominal cavities, reduced abdominal musculature, more flexible ribcage, and lower fat mass, children are more susceptible to multiorgan injuries than adults following BAT [15]. The clinical assessment of pediatric patients with suspected BAT can be challenging, particularly in preverbal children. Furthermore, the presence of multisystem injuries can divert the clinical focus from the abdomen, further complicating the diagnosis. Consequently, diagnostic imaging plays a critical role in the comprehensive evaluation of pediatric trauma patients [16]. CT is currently the gold-standard imaging modality for evaluating hemodynamically stable patients with BAT. However, CT carries limitations, including exposure to ionizing radiation, the risk of iodinated contrast reactions, and inherent risks associated with travel away from the resuscitation area [9, 17]. CEUS can be performed at the bedside, minimizing the need for patient transport, and obviating radiation exposure. A meta-analysis by *Jannatdoust et al.* demonstrated high diagnostic accuracy of CEUS for detecting solid organ injuries in pediatric BAT, with a sensitivity of 88.5% and a specificity of 98.5% [18]. Given these advantages, CEUS has been widely researched as a potential imaging modality for assessing BAT in hemodynamically stable children [2, 18, 19].

Baseline US and color Doppler US are performed using either a linear or curvilinear transducer to assess solid organ abnormalities and hemoperitoneum. Two separate IV bolus injections of UCA are then administered - the first to evaluate the right kidney, right adrenal gland, and liver, followed by the second to assess the pancreas, left kidney, left adrenal gland, and spleen. The pancreas and each kidney should be imaged first because they exhibit rapid enhancement during the arterial phase. In contrast, the liver and spleen are best visualized during the portal venous and delayed phases (Table 1). Additional injections may be administered if necessary [9, 17, 20].

**Table 1:** CEUS scanning technique and optimal contrast phase

Injection	Optimal contrast phase	Organs Imaged
First	Arterial	Right kidney
	Portal venous & delayed	Right adrenal gland, liver
Second	Arterial	Pancreas, left kidney
	Portal venous & delayed	Left adrenal gland, spleen

### Liver

The liver is the second most frequently injured organ following the spleen in cases of BAT. The common types of liver injuries include lacerations, hematomas, and vascular injuries [16]. The majority of liver injuries occur in the posterosuperior region of the right hepatic lobe, known as the “bare area” of the liver [9].

**Fig. 1:**
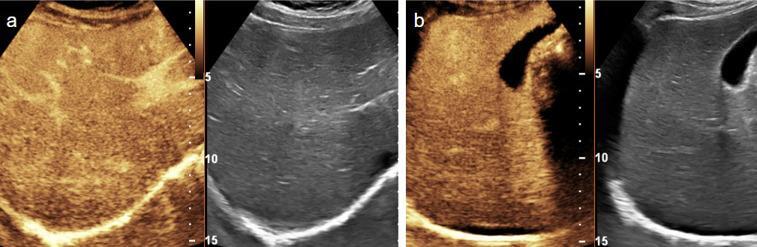
Motor vehicle collision in an 11-year-old girl. (**a**) Transverse and (**b**) sagittal contrast-enhanced ultrasound (CEUS) images with a dual display of contrast (left) and gray-scale (right) mode of the right lobe of liver. CEUS images reveal homogeneous enhancement of hepatic parenchyma with clearly defined hepatic vasculature during the portal-venous phase

The arterial phase of liver enhancement occurs approximately 10–25 s post-injection and lasts for approximately 15 s. Due to the liver’s dual vascular supply, this is followed by the portal venous phase (30–120 s), which is the most effective phase for assessing traumatic injuries. The final phase, known as the sinusoidal and late phase, occurs between 120 and 300 s, extending up to 5 min post-injection. Therefore, the liver is imaged after examination of the ipsilateral kidney. The normal liver parenchyma is homogeneously enhanced with clearly defined vascular structures (Fig. 1) [21].

**Fig. 2:**
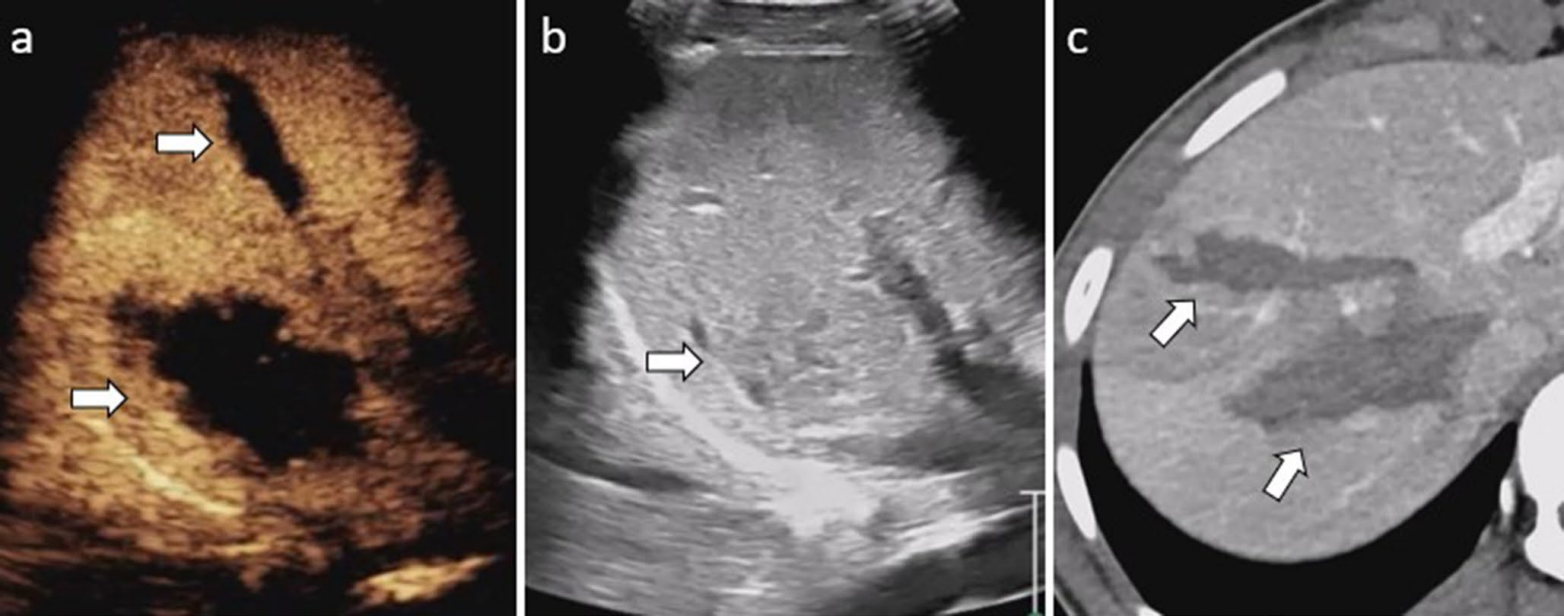
Liver laceration in a pediatric patient. (**a**) Transverse contrast-enhanced ultrasound (CEUS) of the liver reveals irregular hypoenhancing lacerations (arrows) of the liver in segments 5 and 6. (**b**) Transverse gray-scale US of the right hepatic lobe demonstrates a subtle decrease in parenchymal echogenicity in the corresponding region (arrow). (**c**) Axial intravenous-contrast-enhanced CT image confirms the hepatic laceration (arrows)

During baseline US, lesions may appear as inhomogeneous hyper- or hypoechoic areas in the hepatic parenchyma, with or without well-depicted perihepatic collections [2, 21]. On CEUS, lacerations appear as linear or irregular hypoenhancing areas throughout all phases within the parenchyma, while hematomas are characterized by hypoechoic areas with a subcapsular distribution (Fig. 2) [2, 9, 22]. Active bleeding may appear as a blush of microbubbles within a hepatic lesion or hematoma during the arterial phase followed by contrast pooling in the later phase; however, CEUS has limited sensitivity for detecting active bleeding [21].

### Spleen

The spleen is the most frequently injured organ in BAT. Splenic contusion refers to microscopic parenchymal lesion within the parenchyma. Parenchymal rupture is characterized by laceration or tear while the capsule remains intact and is often associated with intraparenchymal or subcapsular hematomas. Splenic parenchymal-capsular rupture involves a laceration that extends through both the parenchyma and capsule. Hemoperitoneum resulting from splenic injury typically traverses the left paracolic gutter into the pelvic cavity [23].

**Fig. 3:**
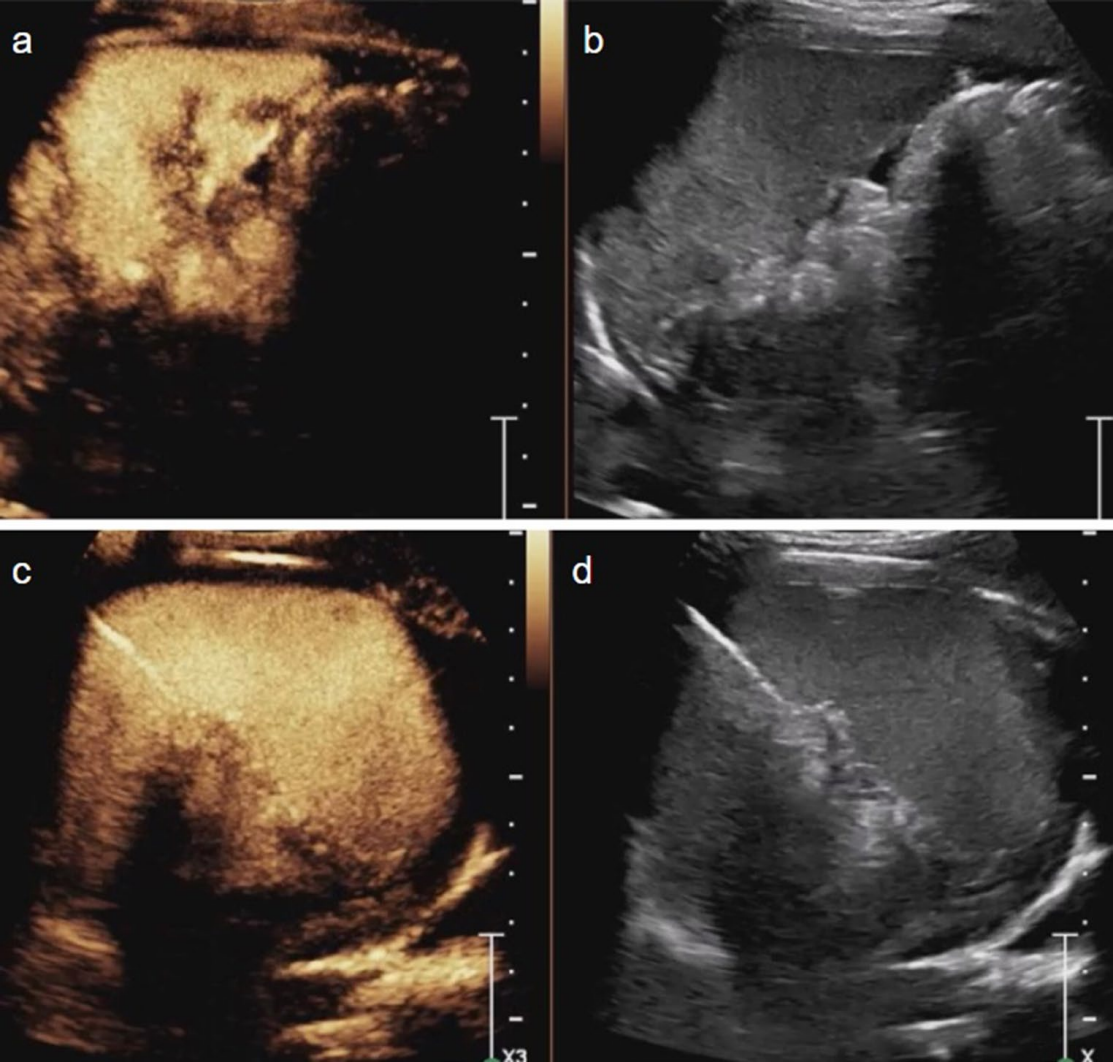
Normal spleen in a pediatric patient. (**a-d**) Transverse contrast-enhanced ultrasound (CEUS) images of the spleen with a dual display of contrast (left) and gray-scale (right) modes at 15 s (**a**,** b**) and 80 s (**c**,** d**) post contrast injection. The spleen reveals arciform enhancement in the arterial phase and homogeneous enhancement in the venous phase in the CEUS images

Similar to CT, during CEUS the splenic parenchyma demonstrates an arciform enhancement pattern in the arterial phase (12–20 s after injection), which is often referred to as a *“zebra”* pattern. The zebra pattern disappears approximately 50 s after the injection. During the venous phase, which lasts 5–7 min, normal splenic parenchymal enhancement is homogeneous (Fig. 3). The spleen should be evaluated during the venous phase to avoid mistaking the normal arciform enhancement for pathology [22, 24].

**Fig. 4:**
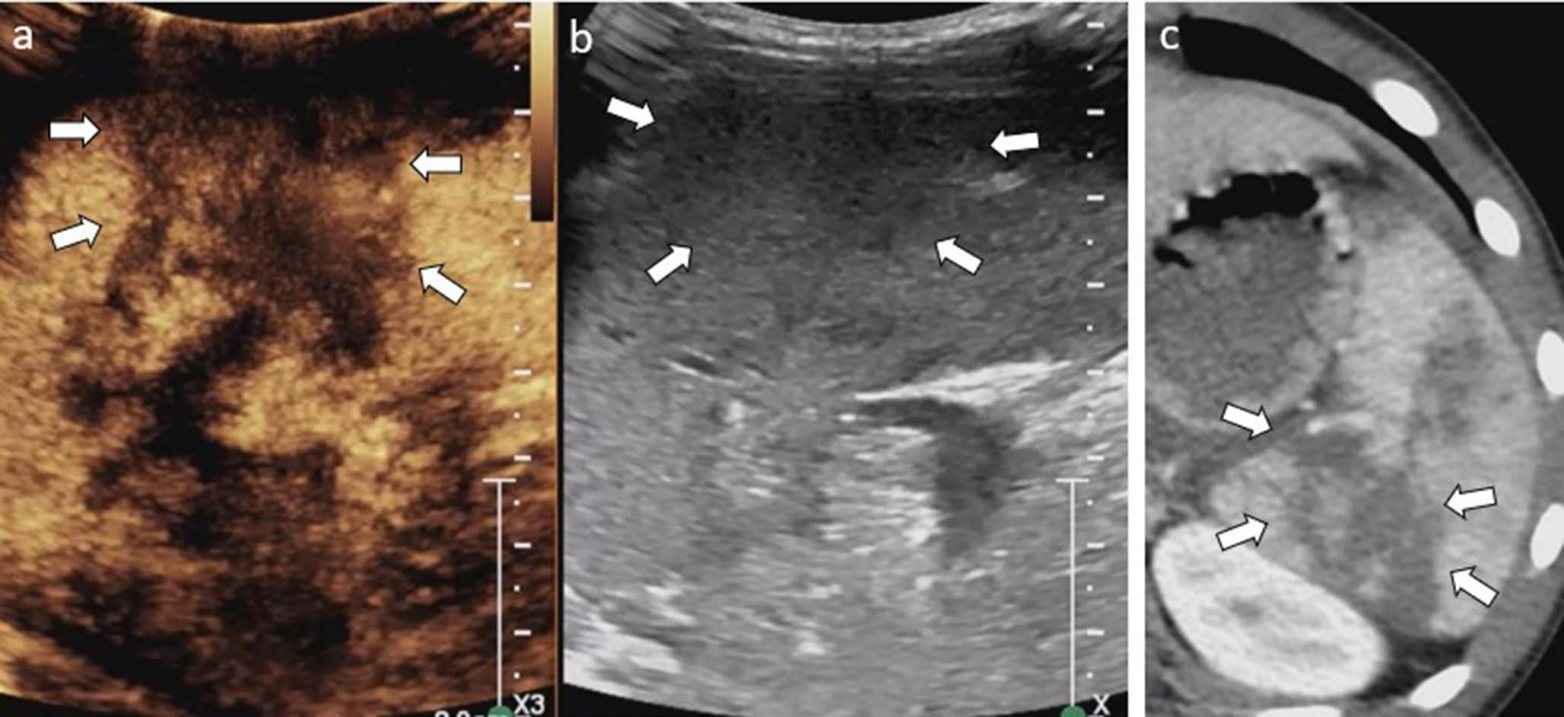
Splenic laceration in a pediatric patient. Sagittal contrast-enhanced ultrasound (CEUS) image of the spleen with a dual display of contrast (**a**) and gray-scale (**b**) modes. CEUS image reveals irregular hypoenhancing lacerations extending from the hilum to the capsule (arrows); however, the gray-scale shows only subtle hypoechogenicity in the subcapsular parenchyma (arrows). (**c**) Axial intravenous contrast-enhanced CT confirms a splenic laceration (arrows)

In the initial gray-scale US, lacerations may be identified as linear or irregular hypoechoic areas, which may extend to the splenic capsule, potentially leading to capsular rupture and hemoperitoneum. However, detecting lacerations on gray-scale US can often be challenging [25]. During CEUS, lacerations appear as linear, branched, or complex hypoenhancing areas, whereas intraparenchymal hematomas appear as non-enhancing areas within the parenchyma with well-defined borders (Fig. 4). Subcapsular hematomas typically exhibit lenticular shapes, conforming to the contour of the splenic capsule without enhancement [9, 22, 26].

### Kidney

The kidney is the third-most commonly injured solid organ in blunt trauma, accounting for approximately 10% of all pediatric BAT cases. Children are more susceptible to renal injury from blunt trauma due to several developmental factors including less perirenal fat, underdeveloped abdominal musculature, and less ossified ribs [27, 28]. As noted previously, CT is the initial modality in cases of severe trauma. However, the European Society of Pediatric Radiology (ESPR) highlighted the role of CEUS in evaluating minor to moderate trauma, serving as an initial diagnostic tool for low pretest likelihood of injury or follow-up assessments [29].

**Fig. 5:**
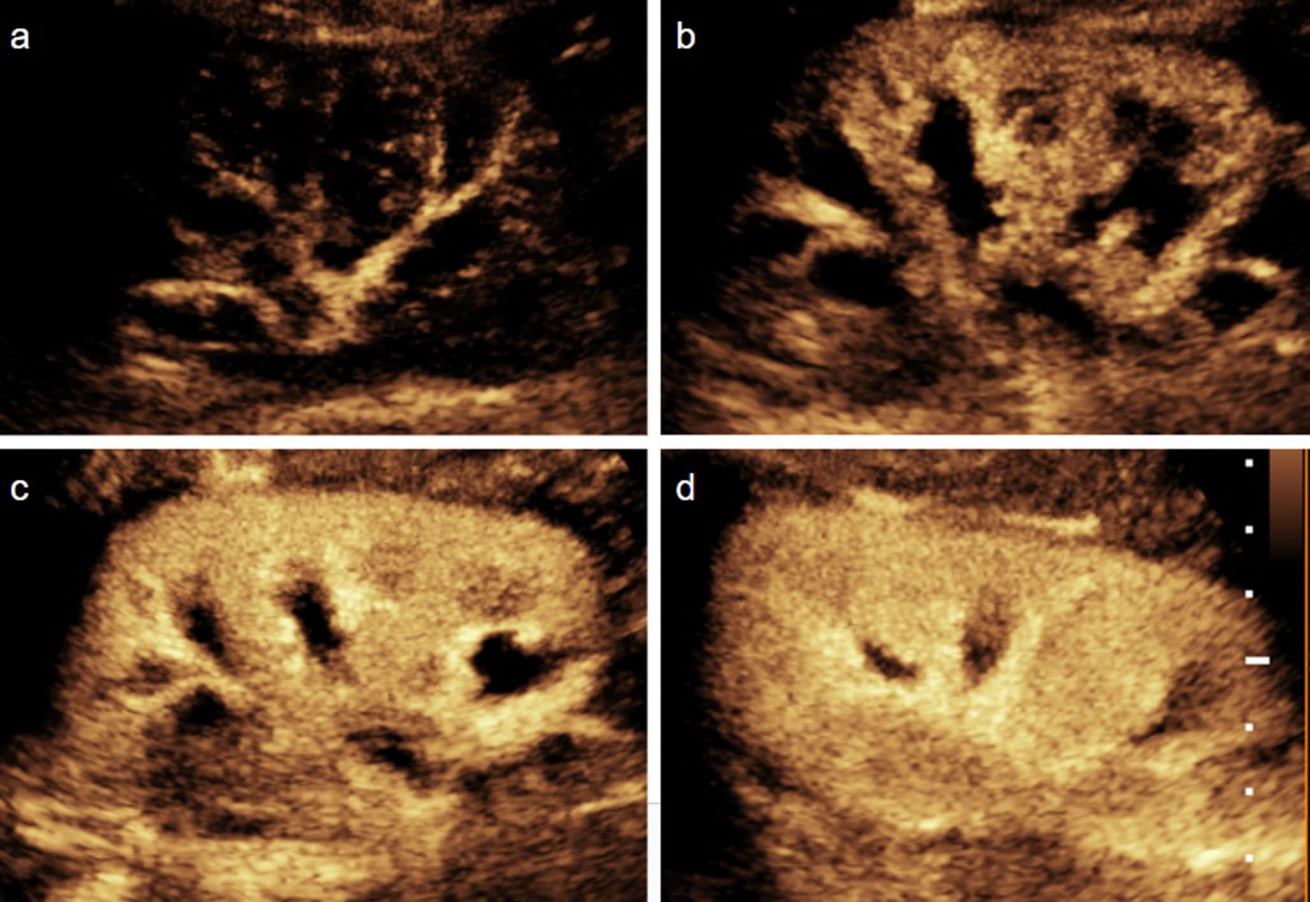
Normal kidney in an 11-year-old girl. Sagittal contrast-enhanced ultrasound (CEUS) image of the left kidney at 10 s (**a**), 15 s (**b**), 30 s (**c**), and 70 s (**d**) post contrast injection. In the first phase, the renal arteries are enhanced first, followed by the renal cortex. In the second phase, the renal medullary pyramids enhance gradually and then the renal parenchyma enhances homogeneously

Two phases of renal parenchyma enhancement have been reported. Following UCA injection, the renal cortex exhibits a more pronounced initial enhancement than the medullary pyramids, typically appearing within the first 15–30 s after injection. Gradual fill-in enhancement of the renal medullary pyramids begins approximately 30 s post-administration. The normal renal parenchyma then exhibits homogeneous enhancement for 2.0–2.5 min, which represents the optimal phase for detecting traumatic injuries (Fig. 5). Unlike contrast-enhanced CT and MRI, UCA remains intravascular; therefore, the excretory phase does not occur [9, 29, 30]. This is important to recognize as CEUS does not play a role in the assessment of renal collecting system injury.

**Fig. 6:**
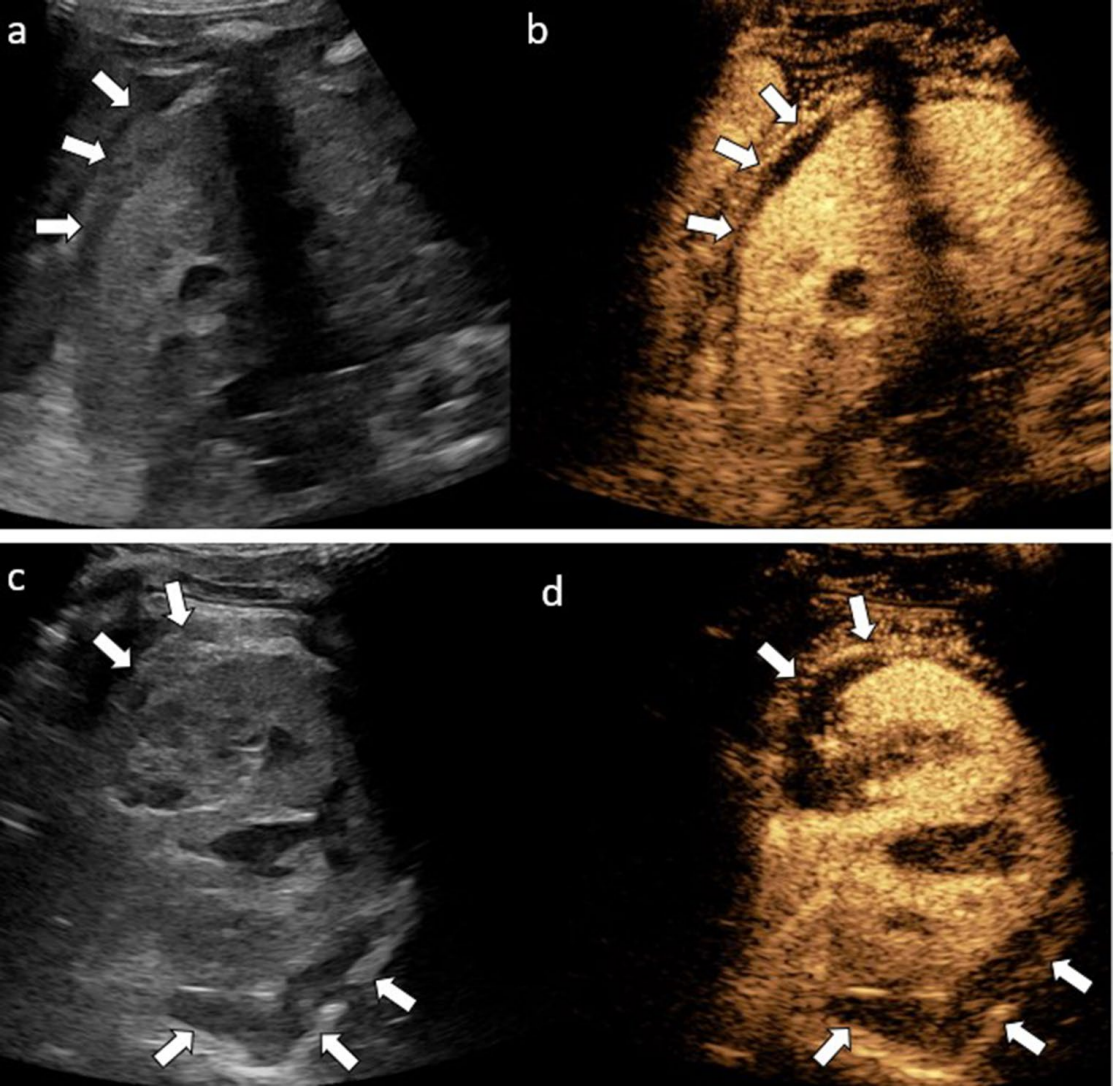
Subcapsular renal hematoma in a pediatric patient. (**a**,** b**) Sagittal and (**c**,** d**) transverse contrast-enhanced ultrasound (CEUS) images of the left kidney with a dual display of gray-scale (left) and contrast (right) modes. The gray-scale US images (**a**,** c**) reveal a hypoechoic subcapsular fluid collection (arrows) around the superior pole of the left kidney. CEUS images (**b**,** d**) demonstrate a large non-enhancing subcapsular hematoma (arrows)

During conventional US, an injured kidney may exhibit a normal appearance or moderate swelling relative to the contralateral, uninjured kidney. During CEUS, traumatic lesions present as well-demarcated, non-enhancing areas within the otherwise homogeneously enhancing renal parenchyma. Extravasation of microbubbles out of the area of injury is indicative of active bleeding. Subcapsular hematomas and perirenal effusions appear as anechoic non-enhancing collections surrounding the kidney, which are easily seen on the background of the well-enhanced kidney (Fig. 6). Vascular injuries may result in the kidney appearing partially or completely hypoechoic [21, 28]. However, the limitations of CEUS include its limited sensitivity in detecting active bleeding and the inability to identify urinary tract injuries because the UCA is not excreted by the kidneys as noted previously [2, 22].

### Pancreas

Pancreatic trauma is less frequent than liver, spleen, and kidney injuries, accounting for only 3–12% of BAT cases. Bicycle handlebar impact is the most common cause of accidental pancreatic injury in children. Pancreatic injuries are notably over-represented in instances of non-accidental trauma [22, 31, 32].

Although evaluation of the pancreas using US is more feasible in pediatric patients than in adults because of less abundant adipose tissue, visualization of pancreas may still be limited by overlying bowel gas and the retroperitoneal location of the pancreas. Consequently, contrast-enhanced CT remains the first-line imaging modality for diagnosing pancreatic injury, as it reveals both specific and non-specific signs of injury.

In conventional US, pancreatic injuries may present as pancreatic enlargement with heterogeneous echotexture, often accompanied by secondary findings such as retroperitoneal hematoma or peripancreatic fluid collections [9, 33]. On CEUS, pancreatic injuries – including laceration, transection, and comminution – appear as non-enhancing or hypoenhancing intraparenchymal defects during both arterial and venous phases. In the study by *Lv*,* Faquin et al.*., CEUS detected blunt pancreatic trauma in 21 of 22 patients with pancreatic injuries confirmed by contrast-enhanced CT. The injured pancreatic regions were characterized by anechoic and/or hypoechoic perfusion defects, whereas conventional US demonstrated pancreatic enlargement with heterogeneous echotexture and indistinct margins in 16 patients [33].

### Adrenal glands

Adrenal trauma following BAT is rare in the pediatric population and is usually associated with ipsilateral or multi-organ injuries. Common causes include motor vehicle and bicycle injuries. The prognosis is generally favorable, and surgical intervention is rarely required except in rare cases of active bleeding [34]. Contrast-enhanced CT is typically used to assess adrenal injury and follow-up. However, CEUS can be useful, particularly in follow-up cases.

Visualization of a normal adrenal gland on US is often difficult; however, adrenal injuries are more readily detectable, appearing as hypoechoic areas within the gland. Adrenal hematomas are typically hypoechoic unless a hemorrhagic pseudocyst, which is a potential complication of adrenal trauma, is present. While the use of CEUS in traumatic adrenal injury is relatively uncommon in clinical practice, prior case reports have demonstrated that CEUS can facilitate the detection of adrenal injuries. These injuries present as lack of enhancement to the gland. Additionally, CEUS improves detection of associated complications such as infarcts, pseudoaneurysms, and contrast extravasation [35–37]. CEUS may also be useful in selected cases requiring follow-up, such as monitoring the resolution of hematomas or excluding evolving vascular complications, as reported by *Rafailidis et al.* [34].

### Scrotal emergencies

Acute scrotum in children and adolescents is a medical or surgical emergency. Gray-scale and color Doppler US are well established as the primary imaging modalities for evaluating acute scrotum [38–43].

### Scrotal injury

Testicular trauma can result in hematocele, hematoma, testicular fracture, or testicular rupture. CEUS offers additional diagnostic information for the assessment of blunt scrotal trauma, helping to clarify the extent of viable testicular tissue [44]. Parenchymal hematomas and contusions appear as hypoperfused areas, whereas hypoperfused bands are associated with testicular fractures or ruptures with disruption of the tunica albuginea [9, 45].

### Testicular torsion

A definitive diagnosis of complete testicular torsion is established when blood flow is absent in the affected testis, with additional findings varying based on symptom duration and degree of spermatic cord rotation [39, 40, 42, 46, 47].

Although initial studies suggested that CEUS does not add diagnostic value to the assessment of complete testicular torsion when compared to color Doppler US [10, 41, 48, 49], recent studies support its role when color Doppler US is equivocal, such as in cases of incomplete torsion. *Fukuzawa et al.* reported a significant reduction in contrast enhancement in the affected testis compared with the contralateral healthy testis in patients with testicular torsion. In contrast, these changes were not observed in the testes of patients presenting with acute scrotum without torsion [46].

Furthermore, a study conducted by *Zou et al.* [50] demonstrated that CEUS provided superior diagnostic accuracy in reclassifying cases of incomplete testicular torsion that had initially been misdiagnosed as complete torsion by color Doppler US, with a 100% accuracy rate in CEUS compared to 66.7% with color Doppler US. An incompletely torsed testicle may retain arterial supply with blood flow on color Doppler US, in which case CEUS can be a valuable tool for demonstrating heterogeneous or abnormal microvascular perfusion within the painful testis [51].

## Ovarian torsion

Ovarian torsion is a gynecological emergency characterized by complete or partial twisting of the vascular pedicle, and pediatric patients are more susceptible to torsion than adults because of relatively long fallopian tubes and suspensory ligaments [52–54].

On gray-scale US, unilateral ovarian enlargement is the most common finding; other findings include increased echogenicity, a solid appearance, peripheral cysts, and atypical ovarian position. Color Doppler US may show reduced or absent blood flow, although this can also be seen in non-twisted pediatric ovaries. Conversely, normal Doppler flow is reported in 57–60% of cases of ovarian torsion [52–54].

CEUS is a promising supplemental modality for the assessment of ovarian parenchymal perfusion, especially when clinical suspicion is high and conventional US findings are equivocal. Diminished or absent enhancement on CEUS suggests ovarian torsion [50, 54]. *Trinci et al.* reported 94.1% sensitivity and 100% specificity of CEUS in a retrospective study of 20 pediatric females with surgical confirmation of torsion, highlighting the additional benefits of improved characterization of ovarian masses and detection of free fluid. The absence of contrast enhancement was detected in 16 of the 17 patients with surgically proven ovarian torsion. The single false-negative case was partial ovarian torsion that exhibited enhancement after contrast administration [54].

### Neonatal and pediatric neuroimaging

*Kastler et al.* first reported the use of CEUS for brain imaging in infants and demonstrated that transfontanellar CEUS identified brain perfusion abnormalities in 10 of 12 examinations, which had not been suspected in conventional US. Furthermore, comparison with MRI showed good or excellent correlation in 10 of the 12 cases [55]. CEUS allows for improved visualization of the cerebral vasculature and microcirculation and better characterization of tissue perfusion. Therefore, CEUS is helpful in evaluating acute neurological conditions such as hypoxic-ischemic encephalopathy (HIE), stroke, and intracranial hemorrhage eliminating the need for sedation or patient transport for MRI.

Similar to other CEUS applications, conventional US is performed prior to CEUS examination. Subsequently, UCA is administered at a dosage of 0.03 mL/kg. A 1-minute cinematic clip is recorded in the mid-coronal plane at the level of the bilateral frontal horns, third ventricle, and basal ganglia using a small-footprint curved array mid-frequency (3–10 MHz) transducer during the wash-in phase (from microbubble arrival to peak enhancement). Anterior-to-posterior sweeps in the coronal plane and lateral sweeps in the sagittal plane should be performed, followed by intermittent 30-second recordings during the wash-out phase, defined as a decline in enhancement after peak enhancement. If necessary, a second UCA injection may be administered to confirm or further characterize initial findings.

### Hypoxic-ischemic encephalopathy

**Fig. 7:**
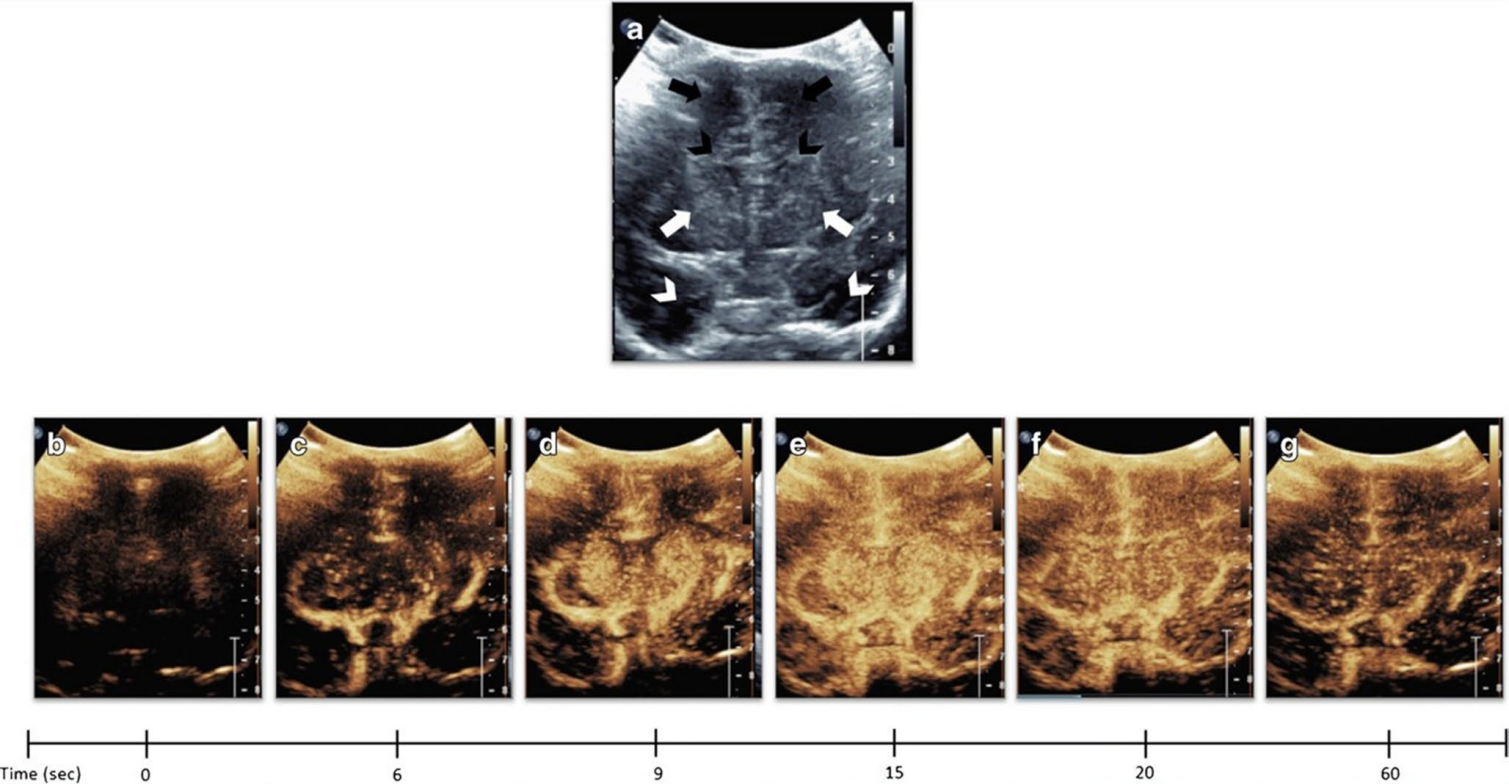
Normal dynamic microbubble wash-in on mid-coronal brain scan in a 1-month-old boy. (**a**) Mid-coronal gray-scale US shows bilateral frontal lobes (black arrows), frontal horns (black arrowheads), basal ganglia (white arrows), and temporal lobes (white arrowheads). (**b-g**) Dynamic microbubble wash-in through the mid-coronal slice through the brain on a contrast-specific mode from the time of injection (time 0) to 1 min. (**c**) Microbubbles flow into the Circle of Willis is partially visualized by 6 s. (**d**) Relatively more prominent enhancement to the basal ganglia with respect to the remainder of the brain is demonstrated by 9 s. (**e**) There is further enhancement of the cortex, with relatively increased enhancement of the basal ganglia by 15 s. (**f**) Contrast wash-out from both the basal ganglia and cortex begins at 20 s and (**g**) continued wash-out is observed at 60 s. Republished from *Hwang M (2019) Introduction to contrast-enhanced ultrasound of the brain in neonates and infants: current understanding and future potential. Pediatr Radiol 49(2):254–262.* 10.1007/s00247-018-4270-1

**Fig. 8:**
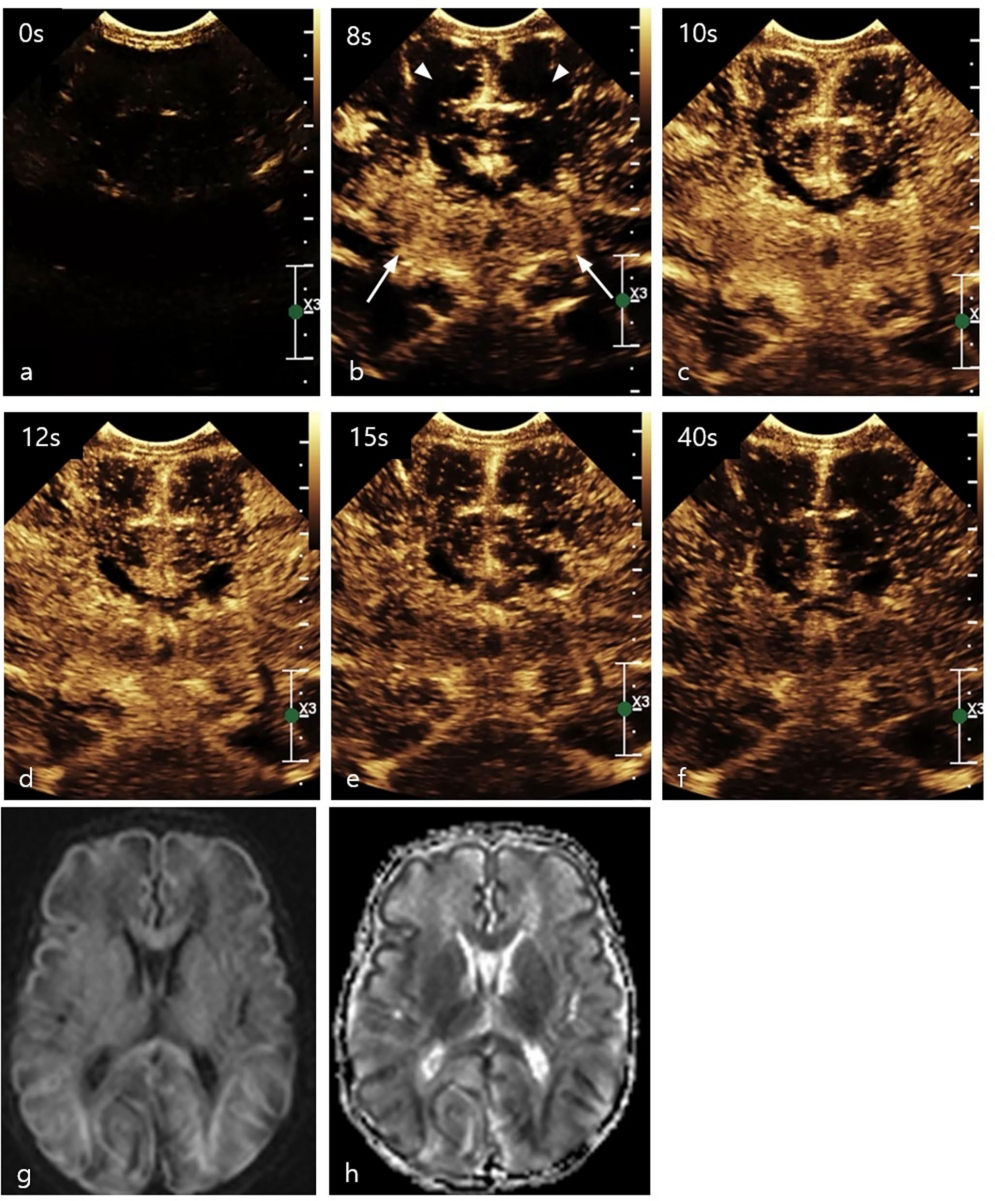
Brain contrast-enhanced ultrasound (CEUS) and MRI scans of hypoxic-ischemic injury in a 21-day-old boy. (**a**) Mid-coronal brain scan before the second contrast administration, with a few residual microbubbles from the first injection. (**b**) Non-homogeneous enhancement in the basal ganglia (arrows) with a paucity of microbubbles in the cortex (arrowheads) is noted at 8 s. (**c)** The same plane at 10 s post-contrast injection demonstrates progressive non-homogeneous enhancement in the basal ganglia and minimal fill-in of contrast in the cortex at 10 s. More avid enhancement of the cortical sulci/leptomeninges as compared to the cortex is noted. (**d)** The same plane at 12 s post-contrast injection demonstrates subtle wash-out of contrast in the basal ganglia, while minimal progressive fill-in of contrast in the cortical gyri more than in the cortex. (**e)** The same plane at 15 s post-contrast injection demonstrates progressive wash-out of contrast from both the cortex and the basal ganglia. **(f)** The same plane at 40 s post-contrast injection demonstrates moderate wash-out of contrast from the cortex and the basal ganglia. (**g)** Diffuse signal abnormalities in the cortical ribbon, white matter, and basal ganglia are noted in diffusion-weighted imaging (DWI) of the mid-axial brain. (**h)** The corresponding apparent diffusion coefficient map demonstrates low signal intensities in the regions affected in the DWI. Republished from *Hwang M*,* Zhang Z*,* Katz J et al. (2022) Brain contrast-enhanced ultrasonography and elastography in infants. Ultrasonography 41(4):633–649.* 10.14366/usg.21224

HIE affects approximately 3 per 1,000 live births in developed countries and can lead to long-term neurodevelopmental dysfunction [56]. In perinatal asphyxia, an initial decrease in blood flow is followed by relative hyperperfusion and release of inflammatory mediators, causing reperfusion injury of the brain tissue [57]. Detection of changes in cerebral blood flow velocity before structural abnormalities appear on CT or MRI allows early diagnosis, which is crucial for optimizing clinical management and may improve prognosis. Although MRI remains the gold standard for diagnosing HIE, preliminary data suggest that CEUS may serve as a complementary tool. CEUS enhances the visualization of the cerebral microvasculature, allowing real-time monitoring of perfusion changes. Perfusion abnormalities in HIE present as focal, multifocal, asymmetric, or symmetrical patterns. While focal and multifocal asymmetric abnormalities are relatively easy to identify, symmetrical abnormalities can be more challenging. *Hwang et al.* introduced a quantitative CEUS approach for screening HIE, using the central-gray-nuclei-to-cortex (GNC) perfusion ratio, defined as a ratio of perfusion in the deep gray nuclei (basal ganglia and thalami) relative to the remaining cerebral cortex. In normal infants, perfusion to the central gray nuclei is greater than that of the cortex, resulting in a GNC ratio slightly greater than 1 (Fig. 7) [58]. In cases of central-pattern HIE, GNC ratios are significantly elevated, whereas a GNC ratio lower than 1 suggests a peripheral pattern of injury (Fig. 8) [57, 59]. These perfusion characteristics can be quantitatively assessed using kinetic parameters derived from the time-intensity curve, including wash-in time, peak enhancement, and area under the curve (AUC).

### Pediatric stroke

The incidence of combined ischemic and hemorrhagic stroke in children is estimated to be up to 13 cases per 100,000 children [56]. MR perfusion imaging is the current standard for evaluating acute stroke; however, CEUS offers a viable alternative for detecting cerebral perfusion defects and monitoring responses to thrombolysis. CEUS provides both qualitative and quantitative assessments of brain perfusion, making it a valuable alternative for critically ill neonates and infants who cannot undergo MRI due to transport limitations or life-support device constraints. Cerebral perfusion findings can vary depending on the timing of the stroke and the characteristics of the affected tissue (tissue at risk vs. injured tissue). CEUS may reveal delayed time to peak in the affected area, slowed UCA transit, and delayed washout rates, indicative of ischemic changes [56].

### Intracranial hemorrhage

CEUS has shown promise in delineating hemorrhagic cores and assessing the extent of cerebral perfusion compromise in cases of intracranial hemorrhage [58]. CEUS can help distinguish between patency and thrombosis in small vessels or areas with slow flow, such as dural venous sinuses [60].

### Bowel: necrotizing enterocolitis

Standard assessment of necrotizing enterocolitis (NEC) using radiographs and conventional US may reveal characteristic findings such as pneumatosis intestinalis, portal venous gas, and pneumoperitoneum [61]. US can also evaluate peristalsis, measure bowel wall thickness, and detect the presence of free fluid. Notably, increased bowel wall thickness and free fluid have been identified as reliable prognostic indicators in multiple single-center studies [62–65]. Color Doppler US has been reported to demonstrate increased perfusion in the affected bowel wall during disease progression. In contrast, cases of bowel necrosis demonstrate absent perfusion in the bowel [66]. CEUS has the potential to assess the vascularity of the suspected bowel wall, including early hyperemia, hypoenhancement, or absence of enhancement in diseased segments of the bowel [67–70]. Consequently, *Mishra et al.*. suggested CEUS as a preferable alternative, particularly in patients undergoing mechanical ventilation [70].

### Limitations

CEUS is recommended for hemodynamically stable patients with isolated low-energy blunt abdominal trauma, in patients with indeterminate or normal CT findings, and for follow-up of conservatively managed traumatic injuries. In contrast, contrast-enhanced CT remains the gold standard in high-energy trauma due to the lower sensitivity of CEUS for detecting vascular active bleeding or urinary tract injury [2, 12, 21].

CEUS possesses several inherent limitations. First, CEUS shares several disadvantages with conventional US, such as operator dependency, interference from bowel gas, body habitus, and need for an acoustic window (e.g., neuroimaging). Second, CEUS offers a limited field of view in comparison to CT and MRI. For example, the dome of the liver, pancreas, or retroperitoneal structures may be less assessable because of their location and bowel gas interposition. Third, adequate training and education are required prior to performing and interpreting CEUS examinations [9, 10, 20, 48, 71].

Despite these limitations, CEUS is a valuable diagnostic tool across various clinical applications given its high diagnostic accuracy, absence of ionizing radiation, the low incidence of contrast-related reactions, and portability. Further multicenter, prospective studies are warranted to establish standardized imaging protocols, interpretation criteria, and clinical workflows to optimize the safe and effective implementation of CEUS.

## Conclusion

CEUS plays an emerging role in the initial workup and follow-up assessment for a variety of traumatic and non-traumatic urgent and emergent conditions in the pediatric population, offering high sensitivity and added value to conventional US. Its potential to reduce unnecessary CT examinations highlights a growing role in pediatric emergency radiology.

## Data Availability

No new data were created or analyzed in this study. Data sharing is not applicable to this article.
